# Prevalence and Risk Factors of Psychiatric Symptoms among Swiss Elite Athletes during the First Lockdown of the COVID-19 Pandemic

**DOI:** 10.3390/ijerph182010780

**Published:** 2021-10-14

**Authors:** Stefan Fröhlich, Christian Imboden, Samuel Iff, Jörg Spörri, Boris B. Quednow, Johannes Scherr, Erich Seifritz, Malte C. Claussen

**Affiliations:** 1 Sports Medical Research Group, Department of Orthopaedics, Balgrist University Hospital, University of Zurich, CH-8008 Zurich, Switzerland; joerg.spoerri@balgrist.ch (J.S.); johannes.scherr@balgrist.ch (J.S.); 2 University Centre for Prevention and Sports Medicine, Department of Orthopaedics, Balgrist University Hospital, University of Zurich, CH-8008 Zurich, Switzerland; 3 Private Clinic Wyss AG, CH-3053 Münchenbuchsee, Switzerland; christian.imboden@pkwyss.ch (C.I.); malte.claussen@pukzh.ch (M.C.C.); 4 Department of Psychiatry, Psychotherapy and Psychosomatics, Psychiatric University Hospital Zurich, University of Zurich, CH-8008 Zurich, Switzerland; samuel.iff@gmail.com (S.I.); quednow@bli.uzh.ch (B.B.Q.); erich.seifritz@bli.uzh.ch (E.S.); 5 Psychiatric Services Grisons, CH-7000 Chur, Switzerland

**Keywords:** competitive sports, SARS-CoV-2, mental problems and disorders, sports psychiatry, sports medicine

## Abstract

The COVID-19 pandemic and the associated first lockdown measures may have had a relevant impact on the mental health of competitive athletes. This study aimed to evaluate the prevalence of various mental health issues in a Swiss elite athletes’ cohort during the first lockdown of the pandemic, and to assess their association with different potential risk factors. Elite athletes from different disciplines were interviewed during the first lockdown in spring 2020 by means of an online questionnaire on symptoms of existing anxieties, depression and sleep disorders, as well as on training circumstances and physical performance before and during the lockdown. Additionally, the economic situation, secondary occupations and current physical health problems were surveyed. A total of 203 (92 female, 111 male) athletes met the inclusion criteria and participated in the survey. Training volume and intensity decreased significantly during lockdown from 3.1 to 2.7 h/day. Financial existential fears increased and were associated with higher training volumes and higher trait anxiety scores. Depressive symptoms and insomnia were present but not exceptionally frequent during the lockdown. Depressive symptoms were associated with higher anxiety scores, higher insomnia severity scores, lower training intensity and worse coping with the measures taken by the authorities against the pandemic. Changes in training and daily habits due to the first lockdown may have affected the mental health of elite athletes. Longitudinal studies should, however, further investigate the long-term effects of the pandemic on mental health.

## 1. Introduction

Mental health issues are common health problems in the general population as well as in competitive sports, as recently stated in an IOC consensus statement [1]. Anxiety and related disorders, major depressive disorders (MDD) and depressive symptoms are frequent in elite athletes and, therefore, particularly relevant [2,3,4,5,6]. Accordingly, MDD in elite sports has received somewhat more attention in recent years than before, as some prominent, tragic cases have come to public attention. Nevertheless, MDD and depressive symptoms often remain a taboo subject in the world of sports.

Knowledge about the exact prevalence of mental health symptoms and disorders in competitive sports is still scarce. The prevalence of generalised anxiety disorder among elite athletes is reported to be between 6% [7] and 14.6% [8]. It is higher in injured athletes, and females are more severely affected than males [1,9]. In general, anxiety symptoms appear to be at least as frequent in athletes as in the general population [1]. For depression, there is no consensus, as the literature provides a wide range between 4% [7] and 68% [10] of athletes with depressive symptoms. Sleep disturbances and disorders are also common in competitive sports [3,11]. Moreover, they represent a major risk factor for various other mental health disorders, such as depression [12,13,14,15], and have a negative effect on physical performance [16,17].

Mental health and physical health should not be considered independently in competitive sports: mental health issues can influence performance, increase the risk of physical injuries and prolong rehabilitation. Injuries, in turn, may affect performance and constitute stress and risk for mental health [1]. Worry, anxiety and psychological stress are also risks to mental health and are associated with the occurrence of psychiatric disorders. Additionally, chronic stress plays an important role in the pathogenesis of anxiety and depression [18]. The COVID-19 pandemic is in itself a stressful situation (fear of getting infected or losing loved ones). Together with the impact of lockdown measures on everyday life it poses a risk to mental health [19].

In Switzerland, the exceptional situation imposed by the Federal Council in the fight against the COVID 19 pandemic, commonly known as the first “lockdown”, lasted from 17 March to 10 May 2020. The lockdown included the closure of shops, public facilities (e.g., restaurants, universities, leisure and sports facilities) and people were advised to stay at home. Individual sportive activities were still possible during the lockdown, although all sport facilities were closed until the 11 May 2020. Thus, such measures represented a break in the lives of athletes, as, from one day to the next, massive restrictions were implemented with regard to their daily habits [20].

In various studies all around the world, impaired mental health and mental health symptoms, e.g., insomnia, increased anxiety levels, fears and depressive symptoms have been reported during the first lockdown [21,22,23]. An increase in pre-existing symptoms has been described as well as new psychiatric symptoms in people experiencing stress, anxiety or grief as a result of the pandemic [24]. Social distancing and quarantine can have an additional detrimental impact on mental health [25]. Elite athletes have been faced with specific restrictions in their daily habits imposed by lockdown measures, some of which go beyond those of the general public. They were even confronted with the postponement of the 2020 Summer Olympics, leading to increased stress and uncertainty as many athletes’ plans and schedules were disrupted [26]. Sports competitions abruptly stopped when COVID-19 cases were detected. Daily training routines that had been established over years were suddenly no longer possible. Depending on their sport (i.e., team vs. individual sports, summer vs. winter sports) and the season in which the lockdown was imposed, the routines of athletes were affected differently. An increased mental health burden in elite football players and athletes of other disciplines has already been described during the lockdown period in spring 2020 [20,27,28,29,30].

Particularly drastic for the athletes was the loss of income that some of them suffered as a result of not being able to pursue their usual sporting activities during the first lockdown. Financial concern has thus been described as a major stressor for athletes during the pandemic [29]. Such a stressor and the accompanying uncertainty about the future can increase the risk of more serious mental health conditions [25]. It is reasonable to assume that financial concerns can also lead to financial existential fears. Therefore, this study also intends to focus on the existence of such existential fears among athletes.

It can be assumed that all of these unintended and unfavourable sudden changes promoted the emergence of anxieties and worries about the future as well as other mental health issues. Furthermore, pre-existing subclinical mental health problems may have increased and become clinically relevant due to the stress caused by the lockdown measures.

Various other risk factors to the mental health of athletes have been identified [31]. Some of them, such as lack of social support, are considered to be particularly prevalent during an exceptional situation such as the pandemic lockdown.

Therefore, the objective of this study was (1) to investigate the prevalence of mental health symptoms such as worries and fears, depressive symptoms and sleep disturbances among Swiss elite athletes of different sports during the first lockdown of the COVID-19 pandemic, and (2) to assess their association with different potential risk factors such as changed training habits, physical performance and other mental health issues during this challenging period.

## 2. Methods

### 2.1. Study Design

The present cross-sectional survey was part of a larger project addressing the mental health of elite athletes during the COVID-19 pandemic. Psychological stress and mental health problems, as well as training habits, subjective physical performance and different aspects of physical health were investigated.

### 2.2. Setting

In spring 2020, during the first lockdown due to the COVID-19-pandemic, an online questionnaire was distributed to top athletes of various sports in Switzerland. For team sports, the distribution was performed via the sports clubs playing in the highest or second highest Swiss national league, for individual sports via the respective national sports associations in Switzerland. Recruitment started on the 25 April 2020 and ended on the 25 May 2020.

### 2.3. Participants

Participants in the study were a random sample of adult elite athletes in Switzerland. All participants were exposed to the lockdown due to COVID-19. Excluded were athletes with incomplete baseline data or not participating in Olympic sports or in sports recognized by the International Olympic Committee (IOC). Athletes were not compensated for participation in this study.

### 2.4. Ethics

This study was reviewed by the local ethics committee and judged not to fall under the scope of the Human Research Act (HRA) by a declaration of non-responsibility (KEK-ZH-NR: Req-2020-00408).

### 2.5. Variables

The collected baseline data included age, sex, type of sport, volume and intensity of training (during and before the lockdown), subjective current physical performance (during and before the lockdown), and secondary occupations. In addition, athletes were asked whether they were fully professional, i.e., whether they were earning sufficient income for their livelihood through sport at the time the study was conducted, without being dependent on an additional income. Additionally, own COVID infection and personal quarantine needs were queried. All participants were also surveyed about depressive symptoms, anxiety, sleep, physical illness and injuries.

In order to screen participants for depressive symptoms, the 9-item depression module of the Patient Health Questionnaire (PHQ-9) was used, which is an established screening tool with a well proven reliability and validity [32]. Anxiety rating was obtained using the German version of the short form of the Spielberger State–Trait Anxiety Inventory (STAI) [33]. The STAI is a validated questionnaire investigating two types of anxiety with 20 (original form) and 10 (short form) items each: trait anxiety as a basic, hardly changeable personal characteristic, and state anxiety as a reaction to current events. In particular, financial existential fears were surveyed for the period before and during the lockdown. Financial existential fears were rated on a scale from 0 (no financial existential fears) to 100 (very severe financial existential fears).

To evaluate sleep disorders, the Insomnia Severity Index (ISI) and extracts from the Pittsburgh sleep quality index were applied, which are validated assessment tools as well [34,35,36]. The Insomnia Severity Index is intended to identify actual disorders, while the parts of the Pittsburgh sleep quality index that were used aim to determine precise parameters such as the length of time it takes to fall asleep and the duration of sleep. In addition, participants were asked on a scale from 0 (“not at all”) to 100 (“very well”) how well they, subjectively, were coping with the officially ordered lockdown measures and whether they were worried about continuing their sporting career.

In addition, current health issues such as injuries or illnesses were recorded using the Oslo Sports Trauma Research Centre (OSTRC) questionnaire on health problems to investigate their association with existing mental health problems [37,38,39]. From the OSTRC questionnaire, the first question determined whether there was an injury or illness. The questionnaires were offered in German and French to allow as many athletes as possible to participate in their native language.

Athletes who showed at least moderate depression symptoms or who expressed suicidal thoughts in the questionnaire were contacted directly by the authors. Personal contact details were provided and an offer was made to refer them to professional help.

### 2.6. Statistical Methods

Baseline data was expressed as mean ± SD and by means of frequency tables for categorical data. To compare demographic data between groups, χ^2^ test was used for tables with more than four fields, one-way analysis of variance for continuous variables, and Kruskal–Wallis rank test for variables nonmetric variables. Differences between the groups were considered significant at *p* < 0.05. For financial existential fears, we fitted a general least squares random-effects model [40] using the value during the lockdown as the dependent variable, previous fears as the independent variable and age, gender, sports category, injury/illness, sufficient income, additional occupation, subjective coping with lockdown measures, and training/performance parameters as covariates. In an attempt to adjust for psychiatric measures, we added the results of PHQ-9, ISI, trait and state anxiety. For depressive symptoms, we fitted the general least squares random-effects model using the PHQ-9 score as the dependent variable and age, gender, sports category, injury/illness, sufficient income, additional occupation, subjective coping with lockdown measures, training/performance parameters, ISI, trait and state anxiety, as well as financial existential fears and worries of career as independent variables. All explanatory variables that had an association with the independent variables at *p* < 0.20 in the univariable analyses were included in the multivariable-adjusted analyses. Using a stepwise backward elimination process, the least significant variables were then removed from the base model. Only variables with *p* < 0.05 remained in the final parsimonious model. Stata Statistical Software (Release 13. College Station, TX, USA) was used to analyse the data.

## 3. Results

### 3.1. Participants

A total of 232 athletes were eligible for the study and answered the questionnaire. Two participants were excluded as they were not performing Olympic or IOC-recognized sports (sports: non-competitive dance, mixed martial arts). Twenty-seven athletes were excluded because they were under 18 years of age at the beginning of the study. Twenty-nine athletes registered outside the inclusion period between 25 April 2020 and 25 May 2020 and were therefore also excluded. Two hundred and three eligible subjects were analysed in the final dataset.

### 3.2. Participant Characteristics

The average age was 24.0 ± 5.2 years. Forty-five percent (*n* = 92) of the participating athletes were female. One hundred and five athletes (52%) competed in summer sports and 98 (48%) in winter sports; 142 (70%) in individual sports and 61 (30%) in team sports. Overall, 60% of the athletes had an additional occupation (*n* = 122) where they worked or studied at least 50% full-time equivalent (FTE) alongside their sport. Those who had an additional job reported to work on average 67.3% ± 28.3% FTE, and 106 of the participating athletes (53%) reported having sufficient income from sport to live on. Three subjects (1%) have had a positive SARS-CoV-2 test and 15 subjects (5%) have had to quarantine themselves for 11.2 ± 5.8 days. Detailed baseline characteristics are shown in Table 1.

In general, there was a well-balanced distribution between female and male athletes and between winter and summer sports. Both individual and team sports were also represented in large numbers. The exact distribution across different sports is shown in Table 2. Of the 15 current Olympic winter sports, 11 (73%) are represented in this study. Of the 37 current Olympic summer sports, 11 (30%) are represented.

#### 3.2.1. Differences between Summer and Winter Sports Athletes

Athletes performing summer sports were more likely to have an additional occupation (71% vs. 48%; *p* = 0.001), although the rate of those with sufficient income from sports did not differ between the groups (48% vs. 58%; *p* = 0.204). Training volume was higher in winter athletes before (winter: 3.4 ± 1.6 h/day; summer: 2.8 ± 1.1 h/day; *p* = 0.003) and during the lockdown (winter: 3.0 ± 1.3 h/day; summer: 2.5 ± 1.1 h/day; *p* = 0.004). Training intensity (% of maximal training intensity) did not differ before the lockdown (winter: 71.5 ± 22.5%; summer: 75.8 ± 16.9%; *p* = 0.132) but was lower in winter athletes during the lockdown (winter: 58.1 ± 22.1%; summer: 64.7 ± 20.8%; *p* = 0.032). Subjective performance capacity (% of maximal performance capacity) did not differ between groups at any time (before lockdown: winter: 76.2 ± 18.6%; summer: 79.7 ± 14.1%; during lockdown: winter: 65.1 ± 17.1%; summer: 70.9 ± 19.6%).

#### 3.2.2. Differences between Team and Individual Athletes

Individual athletes trained more hours per day than team athletes, both before (individual: 3.3 ± 1.5 h/day; team: 2.6 ± 1.2 h/day; *p* = 0.001) and during the lockdown (individual: 2.9 ± 1.3 h/day; team: 2.2 ± 1.0 h/day; *p* < 0.001). Maximal subjective physical capacity was higher in team athletes before (team: 82.0 ± 14.2%; individual: 76.2 ± 17.2%; *p* = 0.023) but not during the lockdown (team: 66.8 ± 18.3%; individual: 68.7 ± 18.8%; *p* = 0.506). With regard to the other parameters mentioned above, there were no significant differences between team and individual athletes.

#### 3.2.3. Changes in Training Habits and Physical Performance

Before the lockdown, athletes trained on average 3.1 (±1.4) hours a day but during the lockdown this was only 2.7 (±1.2) hours a day. This difference was statistically significant (*p* = 0.003). Training intensity dropped from 73.7% (±19.9%) to 61.5% (±21.7%) of their maximum training intensity (*p* < 0.001). There were no significant differences in changes in training habits between winter and summer sports, or between team and individual sports, respectively. Subjective physical performance decreased from 78.0% (±16.5%) to 68.1% (±18.6%) of maximum physical performance capacity (*p* < 0.001). Team sports showed a significantly higher decrease in subjective physical performance (−7.7 ± 21.1%) than individual sports (−15.2 ± 20.8%; *p* = 0.020), whereas there was no significant difference in the decrease of the subjective physical performance between summer and winter sports.

#### 3.2.4. Financial Existential Fears

Participants rated their financial existential fears before the pandemic with a score of 14.5 (±21.5) out of 100, and during the lockdown with a score of 22.3 (±26.9) (*p* = 0.001). In univariate analysis, the financial existential fears during the lockdown were significantly associated with pre-existing financial existential fears before the pandemic (*p* < 0.001). Athletes performing in winter sports had more financial existential fears than those performing in summer sports (*p* = 0.016). Team sports were associated with fewer financial existential fears than individual sports (*p* = 0.011). Athletes with an additional occupation had fewer financial existential fears (*p* < 0.001). Higher PHQ-9 (*p* = 0.001), state (*p* < 0.001) and trait (*p* < 0.001) anxiety scores were associated with more financial existential fears, as were higher training volume (*p* < 0.001), lower training intensity (*p* = 0.025) and poorer coping with the COVID measures implemented by the authorities (*p* < 0.001). According to multivariate regression, only the pre-existing financial existential fears (*p* < 0.001), a higher trait anxiety score (*p* = 0.021) and a higher training volume (*p* < 0.001) were significantly associated with current financial existential fears during the lockdown (see Table 3), all together explaining 64.6% of the variance in financial existential fears (R^2^ = 0.646; *p* = 0.391).

#### 3.2.5. State–Trait Anxiety

Trait anxiety was present during lockdown with a value of 27.2 ± 11.0, and state anxiety with a value of 29.2 ± 11.4 of maximally 80 points. Both did not differ between summer and winter sports (state: *p* = 0.247; trait: *p* = 0.340) or between team and individual sports (state: *p* = 0.361; trait: *p* = 0.246), respectively.

#### 3.2.6. Depressive Symptoms

The PHQ-9 questionnaire showed a mean value of 4.5 (±3.2). Eleven subjects (6%) had a value between 10 and 14, which represents moderate depression severity. Two subjects (1%) had a value between 15 and 19, which represents moderate-to-severe depression. None of the participants showed a higher value representing severe depression. Univariate analysis showed a significant association between a higher PHQ-9 score and multiple factors: female gender (*p* = 0.007), a higher state (*p* < 0.001) and trait (*p* < 0.001) anxiety score, a higher insomnia severity index score (*p* < 0.001), more financial existential fears (*p* = 0.001), a lower training volume (*p* = 0.007) and lower training intensity (*p* = 0.008) during the lockdown, a subjectively lower performance capacity (*p* < 0.001) and worse coping with the measures implemented by the authorities (*p* < 0.001). However, according to multivariate regression analysis, only state (*p* = 0.004) and trait (*p* < 0.001) anxiety scores, insomnia severity index score (*p* = 0.013), lower training intensity (*p* = 0.027) and worse coping with the measures implemented by the authorities (*p* < 0.001) were significantly associated with a higher PHQ-9 score (see Table 4). All together they explained 64.5% of the variance in depressive symptoms (R^2^ = 0.645; *p* = 0.156). Additionally, in multivariate regression, an additional occupation was significantly associated with a higher PHQ-9 score. All of these associations, although statistically significant, were not based on a strong effect. There was no significant difference between the mean PHQ-9 score of individual athletes (4.3 ± 2.9) and team sport athletes (5.0 ± 3.6; *p* = 0.169).

#### 3.2.7. Sleep Time and Subjective Sleep Quality

Participants spent an average of 8.8 ± 1.0 h per night in bed. The time between falling asleep and getting up was reported as 8.4 ± 1.0 h. The effective sleep time was reported as an average of 7.9 ± 1.1 h per night. Individual athletes slept significantly longer than team sports athletes (effective sleep time: 8.1 h vs. 7.7 h; *p* = 0.017). Participants with an additional occupation slept less than those without (effective sleep time: 7.7 h vs. 8.2 h; *p* = 0.002). The mean insomnia severity index score was 5.4 ± 4.1 on the scale from 0 to 28. Forty-seven subjects (24%) had a score between 8 and 14, which means a sub-threshold insomnia, and seven (4%) had a score between 15 and 21, which means a moderate insomnia. None of the participants had a score indicating a severe insomnia. The scores did not differ significantly between summer and winter sports (*p* = 0.149) or between team and individual sports (*p* = 0.334), respectively.

## 4. Discussion

The main findings of the study were: (1) financial existential fears were significantly higher during lockdown; (2) financial existential fears were mainly present among individual athletes, less so among team sports athletes; (3) financial existential fears during lockdown were associated with higher training volumes, higher trait anxiety scores and with pre-existing financial existential fears before the pandemic; (4) depressive symptoms and insomnia were not exceptionally common; (5) depressive symptoms were associated with higher anxiety scores, higher ISI scores, lower training intensity and worse coping with the measures implemented by the authorities; (6) sub-threshold or moderate insomnia was prevalent.

As shown in this study, financial existential fears increased during the first lockdown. This might be explained by the fact that cancelled competitions deprived athletes of the public stage and of direct income, such as prize money. It is difficult to find an explanation for the greater financial existential fears among winter sports athletes as they had almost finished their season before the first lockdown, and, thus, their actual income was only marginally affected. From a pure speculative point of view, a potential reason might be the fact that in our sample, winter sports athletes had significantly fewer additional occupations. This would leave them without additional income to compensate for reduced income from sport. Another hypothetical explanation could be that some athletes performed below their expectations last season, which may have led to financial concerns for the future.

Athletes from team sports exhibited fewer financial existential fears, although they showed a greater loss in terms of their performance capacity. Other authors found generally more psychological distress in individual sports athletes without asking for financial existential fears in particular [41]. However, the fact that team sports athletes trained less might give them more opportunities to work in additional jobs. The same explanation may apply for the finding that higher training volume during the lockdown is associated with more financial existential fears: the less they train the more time they have to work or to study. The lower performance capacity in team sports could be explained by the fact that in team sports, creating regular training situations was impossible during the lockdown.

Our results show a rather low rate of depressive symptoms in the studied sample. Although there were athletes indicating moderate or moderate-to-severe depressive symptoms, their number was relatively low (7%). In contrast to our findings, Gouttebarge and colleagues found moderate and moderate-to-severe depression levels in 12.9 (male) to 21.6% (female) of professional football players during the first COVID-19 lockdown period [27]. Pensgaard et al. found depressive symptoms in 22.3% of a Norwegian sample of elite athletes in summer 2020 but did not report the symptom severity [29]. In a South African sample of elite and semi-elite athletes, almost half of them reported feeling depressed during the lockdown in spring 2020 [28]. Another study found exactly the same rate (7%) of people with at least moderate depression as we did in the Swiss general population during the COVID-19 pandemic [42]. However, the number of participants from Switzerland was only 57.

While it has been reported that depressive symptoms are more prevalent in individual sport athletes than in team sport athletes [43,44], this is not supported by our data. The higher prevalence of depression in females is well known and can be confirmed in our sample [1,45]. In particular, female athletes with low socio-economic status and poor training conditions seem to suffer from especially severe psychological symptoms during lockdown [20]. A robust body of evidence shows strong associations between depression and anxiety [46] as well as depression and sleep disturbance [47]. Thus, the corresponding correlations found in this study are entirely compatible with the current state of knowledge. The association of depressive symptoms with lower training intensity and personal coping with the measures taken by the authorities is also plausible. The time freed up by less training can lead to doubts and perhaps a crisis of purpose. Poor coping with the lockdown measures is in itself a stressor. Since changes in training habits enforced by the lockdown seem to be a psychological stressor, normal training should be maintained as usual, as far as the measures allow. A negative correlation between physical activity level and mental health symptoms during the pandemic has also been shown before [48]. Combining workouts with online meetings (for team and individual athletes) could also be an opportunity to support mental health during such periods, as social support has been shown to be a protective factor for mental health in athletes in general [31].

Twenty-eight percent of the athletes showed a sub-threshold or moderate insomnia in ISI. In accordance to our results, Pillay et al. found sleep not to be restful in 25–30% of their sample of *n* = 692 South African elite to semi-elite athletes during lockdown [28]. An Italian study found sleep disturbances (self-reported: yes or no) in 42.2% of the general population during the last 2 weeks of the first lockdown in 2020 [22], while, generally in Europe, approximately 6% of the general population suffer from chronic insomnia [49]. Thus, our results seem to show a higher than normal rate of sleep problems during the lockdown period, which is in line with the results of the aforementioned studies.

## 5. Study Limitations

Firstly, the type of data collection used (online questionnaires) can only provide a description of various mental health symptoms. A clinical diagnosis of mental disorders cannot be made and no conclusions can be drawn as to whether psychiatric treatment is indicated. Secondly, data concerning the pre-lockdown period were queried retrospectively, which increases the likelihood of biased information. Thirdly, the recruited sample may not be entirely representative of elite athletes in Switzerland, and external validity could be limited as selection bias may have occurred. Fourth, for a better interpretation of our results it would have been helpful to have a control sample with the same variables during normal times. Fifth, the number of participants limits the finding of highly significant observations regarding differences between subgroups. However, as it is difficult to find larger collectives of elite athletes, this is nevertheless one of the larger studies reporting on the mental health of athletes during the COVID-19 pandemic.

## 6. Conclusions

Changes in training and daily habits due to the first lockdown of the pandemic may have affected the mental health of elite athletes. The initial lockdown measures represented a significant break in the daily routines of elite athletes, but prolonged, albeit less severe, restrictions during the pandemic may have a different psychiatric impact. Therefore, although the present study has not yet demonstrated alarmingly high rates of mental health symptoms, longitudinal studies during the pandemic should further investigate these developments as well as potential long-term effects so that appropriate therapeutic strategies can be developed if necessary. Such longitudinal studies could also be used to assess the effectiveness of preventive measures. Appropriate preventive measures could be, for example, psychoeducational measures in sports associations or direct access for athletes to the relevant professionals mediated by the association, as well as efforts to provide as normal training conditions as possible. Wherever possible, outpatient psychiatric consultations should additionally be offered virtually or by phone [50,51] to ensure that everyone has access to psychiatric care despite quarantine rules. In this context, the WHO has also launched an initiative to improve access to mental health services for the general population worldwide [52].

## Figures and Tables

**Table 1:** Baseline characteristics.

	Unit	Overall	All Sports		Winter Sports		Summer Sports	
			Female	Male	Female	Male	Female	Male
Total number of athletes	#	203	92	111	43	55	49	56
Athletes with additional occupation	%	60.1	65.2	55.9	51.2	45.5	77.6	66.1
Workload in additional occupation (% of full pensum)	mean	67.3	65.2	69.3	55.6	62.3	72.3	74.9
	SD	28.2	27.9	28.3	26.4	29.9	26.9	25.7
Athletes with sufficient income from sport	%	52.2	52.2	52.3	67.4	49.1	38.8	55.4
Mean training volume (during lockdown) (h/day)	mean	2.7	2.6	2.8	3	2.9	2.3	2.6
	SD	1.2	1.2	1.3	1.3	1.4	1	1.2
Mean training volume (before lockdown) (h/day)	mean	3.1	3.3	3	3.8	3.1	2.8	2.8
	SD	1.4	1.4	1.4	1.5	1.6	1.2	1.1
Ratio training volume during/before lockdown	%	87.1	80.4	93.3	80.5	93	80.2	93.6

#: absolute number of athletes; %: percentage proportion of athletes; SD: standard deviation.

**Table 2:** Sport disciplines of the participants.

Summer Sports	Overall	Female	Male
Artistic swimming	0	0	0
Athletics	14	9	5
Basketball	0	0	0
Boxing	1	0	1
Cycling	28	11	17
Dancing	0	0	0
Equestrian	1	1	0
Fencing	1	1	0
Floorball	4	0	4
Football	24	10	14
Handball	5	0	5
Judo	7	4	3
Orienteering	2	1	1
Shooting	1	1	0
Swimming	2	2	0
Triathlon	15	9	6
**Total**	105	49	56
**Winter Sports**	**Overall**	**Female**	**Male**
Alpine skiing	18	10	8
Biathlon	6	3	3
Bobsleigh	20	5	15
Cross-country skiing	8	2	6
Freestyle skiing	9	5	4
Ice hockey	8	0	8
Nordic combined	1	0	1
Skeleton	2	2	0
Ski jumping	4	1	3
Snowboarding	21	15	6
Speed skating	1	0	1
**Total**	98	43	55

Data are expressed as the number of participating athletes.

**Table 3:** Multivariate regression of financial existential fears.

Independent Variable	Univariate		Multivariate	
	Beta Coefficient	*p*-Value	Beta Coefficient	*p*-Value
Financial existential fears before lockdown	0.896 ***	<0.001	0.785 ***	<0.001
Age	0.469	0.195	0.337	0.133
Winter sport (vs. Summer sport)	9.166 *	0.016	4.262	0.081
Team sport (vs. Individual sport)	−10.533 *	0.011		
Additional occupation	−16.246 ***	<0.001		
PHQ-9 sum	1.922 **	0.001		
Trait anxiety	0.696 ***	<0.001	0.267 *	0.021
State anxiety	0.562 ***	<0.001		
Training activity (hours/day)	6.171 ***	<0.001	5.841 ***	<0.001
Training intensity (% of maximal intensity)	−0.194 *	0.025	−0.102	0.090
Coping with COVID measures	−0.389 ***	<0.001	−0.115	0.053

PHQ-9: Patient Health Questionnaire 9. * *p* < 0.05, ** *p* < 0.01, *** *p* < 0.001.

**Table 4:** Multivariate regression of PHQ-9 questionnaire sum.

Independent Variable	Univariate		Multivariate	
	Beta Coefficient	*p*-Value	Beta Coefficient	*p*-Value
Female (vs. male)	1.226 **	0.007	0.518	0.077
Additional occupation	0.834	0.074	0.607 *	0.037
Trait anxiety	0.214 ***	<0.001	0.123 ***	<0.001
State anxiety	0.191 ***	<0.001	0.060 **	0.004
ISI sum	0.343 ***	<0.001	0.093 *	0.013
Financial existential fears during lockdown	0.027 **	0.001		
Training activity (hours/day)	−0.504 **	0.007		
Training intensity (% of maximal intensity)	−0.028 **	0.008	−0.015 *	0.027
Coping with COVID measures	−0.057 ***	<0.001	−0.028 ***	<0.001
Subjective performance capacity(% of maximal capacity)	−0.049 ***	<0.001		

ISI: Insomnia Severity Index. * *p* < 0.05, ** *p* < 0.01, *** *p* < 0.001.

## Data Availability

The data presented in this study are available on request from the corresponding author. The data are not publicly available due to privacy reasons.
